# Impairments in sleep and brain molecular clearance in people with cognitive deterioration and biological evidence of AD: a report of four cases

**DOI:** 10.1186/s12883-023-03460-8

**Published:** 2023-11-22

**Authors:** Mariateresa Buongiorno, Esther Granell, Giovanni Caruana, Gemma Sansa, Yolanda Vives-Gilabert, Natalia Cullell, Jessica Molina-Seguin, Marta Almeria, Cristina Artero, Gonzalo Sánchez-Benavides, Nicola J Ray, Sonia A.L. Correa, Jerzy Krupinski

**Affiliations:** 1 Department of Neurology, F.Ass. Mútua Terrassa, Terrassa (Barcelona), Spain; 2 Radiology Department, UDIAT-Parc Taulí Sabadell, Sabadell (Barcelona),, Spain; 3 Department of Radiology, F.Ass. Mútua Terrassa, Terrassa (Barcelona), Spain; 4 AdSalutem, Aptima, Mútua Terrassa, Terrassa (Barcelona), Spain; 5 Intelligent Data Analysis Laboratory (IDAL), Department of Electronic Engineering, Universitat de València, Valencia, Spain; 6 Fundació per a Docencia I Recerca, Mútua Terrassa, Terrassa (Barcelona), Spain; 7 Barcelona eta Brain Research Center, Barcelona, Spain; 8 Faculty of Health and Education, Manchester Metropolitan University, 53 Bonsall Street, Manchester, M15 6GX UK

**Keywords:** Glymphatic system, Polysomnography, Gadolinium-based contrast agent, Cognitive function, Obstructive sleep apnea, Prodromal AD

## Abstract

**Background:**

Recent evidence suggests that the failure of the glymphatic system – the brain’s waste clearance system, which is active during sleep – plays a key role in the pathophysiology of Alzheimer’s Disease (AD). Glymphatic function can be investigated using serial MRIs after intrathecal gadobutrol injection. This technique can reveal the health of the glymphatic system, but has not yet been used in participants with cognitive impairment due to AD.

**Case report:**

This report describes the sleep and gadobutrol tracer clearance patterns of four participants diagnosed with mild to moderate cognitive impairment with evidence of AD pathology (pathological levels of Ab and p-tau in cerebrospinal fluid). We performed polysomnography and MRI studies before tracer injection and MRI scans at 1.5-2 h, 5–6 h, and 48 h after injection. Despite participants reporting no sleep problems, polysomnography revealed that all participants had moderate to severe sleep disturbances, including reduced sleep efficiency during the study and obstructive sleep apnea. Severe side-effects related to tracer administration were observed, impeding the completion of the protocol in two participants. Participants who finished the protocol displayed delayed and persistent tracer enrichment in the cortex and white matter, even 48 h after injection. These outcomes have not been observed in previous studies in participants without AD.

**Conclusion:**

The findings suggest that brains with sleep impairment and AD pathology have poor glymphatic function, and therefore cannot clear the contrast tracer efficiently. This is likely to have caused the severe side effects in our participants, that have not been reported in healthy individuals. Our results may therefore represent the only available data acquired with this technique in participants with AD pathology.

**Supplementary Information:**

The online version contains supplementary material available at 10.1186/s12883-023-03460-8.

## Introduction

The anatomical and physiological features of the glial-lymphatic (glymphatic) system have been well-characterised in murine models [1] but is still not well understanded in humans. Recent experimental evidence in rodents has shown that cerebrospinal fluid (CSF) influx into the brain, as well as glymphatic system efficacy in clearing metabolic waste, are increased during non-rapid eye movement (NREM) slow-wave phases of sleep [2–4]. Therefore, disturbances in the duration of NREM sleep phases may cause a reduction in glymphatic system efficiency, resulting in accumulation of amyloid beta (Aβ), tau, and α-synuclein among other proteins involved in the physiopathology of neurodegenerative diseases [5]. As the amount of NREM sleep reduces with age [6], it is important to determine whether disturbances in sleep regulate the clearance of metabolic waste in the human brain.

The first attempt to quantify the effect of sleep deprivation on glymphatic function in-vivo in humans was made recently by Eide and colleagues [7]. They examined the clearance of gadobutrol injected intrathecally, via serial MRIs in two groups of participants. The first group underwent total sleep deprivation for one night, while the other was allowed to sleep freely. The authors found that there were significantly increased tracer levels within the cerebral cortex and white matter after one night sleep deprivation, indicating reduced tracer clearance. This was observed particularly in limbic structures (amygdala, hippocampus, nucleus accumbens, prefrontal cortex, insula and cingulum). Tracer levels clearly declined after 48 h but remained elevated in the sleep deprived group, indicating that clearance is not fully normalized even with subsequent sleep. These important findings are the first direct evidence that sleep has an impact on glymphatic system functioning. Specifically, lack of sleep may compromise the clearance of metabolites and waste proteins in the brain.

To date, there are no studies using intrathecal gadobutrol injection with serial MRI in individuals with protein aggregation-related disorders, such as Alzheimer’s disease (AD). Elucidating the factors associated with sleep disturbances and glymphatic system failure in these participants is of paramount importance if we are to rationally design new treatments that target glymphatic function.

We therefore designed a longitudinal study in cognitively impaired individuals with evidence of AD pathology to explore the association between sleep disturbances and glymphatic system function in these individuals. In the study, participants undertake a polysomnogram along with clinical, cognitive, and genetic (*APOE-e4, AQP4*) explorations. In addition, we planned to measure glymphatic health via intrathecal injection of gadobutrol (Gd) as a contrast medium for subsequent MRI. [7] However, despite following the methods of previous reports precisely, all four participants reported serious side effects. As such, we have adapted the protocol, and will now use intravenous gadolinium as the contrast medium for all future participants. This study is currently ongoing and recruiting.

Since it may not be possible to use intrathecally injected gadolinium in future work in AD and/or those with sleep disturbances, we are reporting the outcomes from our four participants as a case report.

### Case report

We invited participants with memory complaints referred to the Cognition and Behavior Unit at the Department of Neurology, Hospital Universitari MútuaTerrassa, (HUMT), Barcelona, Spain with evidence of pathological levels of amyloid-beta (Aβ) and phosphorylated -tau in CSF and a score of at least 0.5 in the memory domain in the Clinical Dementia Rating (CDR). Objective cognitive performance was assessed using the Mini-mental State Examination (MMSE) and the delayed memory index of the Repeatable Battery for the Assessment of Neuropsychological Status (RBANS) [8]. We used the authorized Catalan/Spanish translation of the MMSE supplied by our institution. CSF levels of Aβ1–42, Aβ1–40, total (t)-tau, and phosphorylated tau at threonine 181 (p-tau) were measured using the Lumipulse essay kits from Fujirebio (Fujirebio Inc. Europe, Gent, Belgium). We determined positivity of AD core biomarkers using local cut off values [9]. Detailed information can be found in Additional file 1.

Participants with mild (T001 and T002) to moderate (T003 and T004) cognitive impairment displayed pathological levels of total Aβ_1−42_, phosphorylated and total tau as well as ratios between Aβ_1−42_/Aβ_1−40_ and T-tau/Aβ_1−42_ (Table 1).

**Table 1:** Demographic and clinical data, scores for the cognitive tests and biomarkers analyses. MMSE: Mini Mental State Examination; RBANS-DMI: Repeatable Battery for the Assessment of Neuropsychological Status-Delayed Memory Index. Protein expression levels of biomarkers in the CSF of participants. Cut-off points to define positivity for AD: Aβ_1−42_ < 638 pg/mL; total (t)-tau > 404 pg/mL; p-tau 181 > 52.1 pg/mL; t-tau/ Aβ_42_ > 0.784; Aβ_1−42_/Aβ_1−40_ < 0.068. [9]

Participants	T001	T002	T003	T004
Age (years)	74	70	73	79
Sex	Male	Female	Female	Male
Education (years)	8	8	8	12
MMSE	21	22	25	15
RBANS-DMI	56	93	48	40
CDR CSF Aβ_1−42_ (pg/mL)	0.5 370	0.5 239	0.5 335	2.0 216
CSF p-tau 181 (pg/mL)	133.8	161.9	101.1	88
CSF T-tau (pg/mL)	734	848	661	568
Aβ_1−42_/Aβ_1−40_	0.046	0.034	0.029	0.03
T-tau/Aβ_1−42_	1.984	3.548	1.862	2.63

### Polysomnography (PSG) and sleep architecture

Participants underwent an overnight PSG at the HUMT sleep unit that included videorecording, electroencephalogram (EEG), electromyography (EMG) electrodes, nasal cannula, thermistor, and pulse-oximeter. Sleep stages and respiratory events were scored according to the American Academy of Sleep Medicine standard criteria (a detailed description of PSG methods can be found in Additional file 1). Despite participants did not report any sleep complaint, all four had sleep disturbances according to the PSG, but with different degrees of severity (Table 2).

Briefly, sleep efficiency was altered in all participants. In particular, participant T003 had very low sleep efficiency (39%; percentage of time in bed spent asleep) and high periodic limb movements during sleep (PLMS) index (a ratio of limb movements over hours of sleep). In fact, this participant slept only 199.5 min in total, of which 30 min was spent in REM sleep (15% of total sleep time). 57 min was spent in N1 sleep; 112 min in N2 and only 0.5 min in N3 stages. Clearly, there was a significant reduction in the amount of time spent in REM and stage N3 (0.3% of total sleep time). Although there were severe disruptions in sleep patterns, these were not caused by obstructive sleep apnea (OSA), as shown by T003’s low apnea/hypopnea (AHI) scores.

Sleep efficiency of the remaining 3 participants was also reduced (66.7%, 66.2% and 61.8%, for T001, T002 and T004, respectively). For comparison, sleep efficiency is shown to be at approx. 85% in healthy individuals of similar age [6]. The observed short sleep latency in patient T001 could be related to the presence of increased sleepiness because of severe sleep obstructive apnea.

T004 spent nearly 70% of sleep time with oxygen saturation below 90% (ST_90_) combined with high AHI index (40.9), indicating severe OSA (Table 2). Although participant T001 was also diagnosed with severe OSA, with an AHI index of 37.9, ST_90,_ the level of desaturation was low (Table 2). Patient T002 showed mild level of apnea, with a decreased percentage of REM sleep and increased duration of NREM-N1 stage (Table 2). The observed OSA might have a relevant impact in sleep architecture. Indeed, we observed an increase in N1 sleep and a decrease in slow wave and REM sleep, that could be explained by sleep fragmentation because of arousals associated to these events.

**Table 2:** Sleep objective and subjective data. Absolute parameter values obtained from a full night sleep polysomnography (PSG) recording, and Pittsburgh Sleep Quality Index (PSQI) [10] scores

PSG Parameters	T001	T002	T003	T004
Sleep Latency (min)	1.8	14.4	40.0	49.5
Total sleep time (min) Sleep efficiency (%)	315.0 66.7	314.5 66.2	199.5 39.0	297.5 61.8
N1%	43.3	17.0	28.6	28.4
N2%	46.5	45.5	56.1	51.4
N3%	5.2	24.3	0.3	10.6
REM %	4.9	13.2	15.0	18.8
PLMS index (events/hr)	2.9	0	26.8	0
AHI (events/hr)	37.9	10.9	8.4	40.9
WASO (min) ST_90_ (%)	155.5 0.6	146.1 0.1	271.7 8.4	134.6 69.7
Pittsburgh Sleep Quality Index (PSQI)	15	6	NA	4

Abbreviations: periodic limb movement during sleep (PLMS); Apnoea/hypopnea index (AHI); rapid eye movement (REM), Non-REM stage 1 (N1); stage 2 (N2) and stage 3 (N3); wakefulness after sleep onset (WASO) and total sleep time with oxygen saturation below 90% (ST_90_).

### MRI acquisition and patterns of tracer enrichment and clearance

After spending the night at the sleep clinic, participants were transferred to the Radiology Department (UDIAT-Parc Taulí Sabadell) for MRI scans and intrathecal injection of the contrast. Following a pre-contrast MRI scan, participants were prepared for CSF collection (5 mL) for biomarker analysis and contrast administration (0.5 ml of 1.0 mmol/ml Gd; Gadovist^R^, Bayer Pharma AG). Correct position of the gauge needle tip in the subarachnoid space between lumbar L3/L4 or L4/L5 interspace was verified by CSF backflow from the puncture needle and CSF was collected before replacing the syringe containing the contrast diluted in 2 ml physiological serum. After the contrast injection, the patient was instructed to rotate once around the body axis on the table and lay down until the second MRI scan was taken 1.5-2 h after injection. The third scan was taken 5–6 h and the last scan was taken 48 h after injection. Participants were laid down until the last MRI acquisition during the first day and allowed to move freely thereafter. Participants T001 and T002 were able to complete all four MRI scans, whereas participants T003 and T004 were able to complete only the first two scans due to severe side effects caused by intrathecal injection of gadobutrol. MRI scans were acquired using a 3T MR scanner (Phillips Ingenia Elition). A standardized MR protocol was used for the acquisition, comprising of high-resolution 3D T1-weighted magnetization-prepared rapid gradient echo imaging sequence for enhanced tissue.

To calculate tracer enrichment in the CSF, a region of interest was manually placed in the cisterna magna on T1-weighted images of each participant (Additional file 1; Figure S1a). The obtained signal intensity was then normalized (i.e., divided by a reference value measured from the posterior part of the orbit (Additional file 1; Figure S1b), where the tracer cannot reach. To calculate tracer enrichment in brain parenchyma, we first segmented the brain using the FreeSurfer software version 7.3.2 (http://surfer.nmr.mgh.harvard.edu/ ). In brief, we used subcortical segmentations and cortical parcellations to investigate the abundance of the tracer within the brain by analyzing the increase of T1 signal intensity at each time point.

**Fig. 1:**
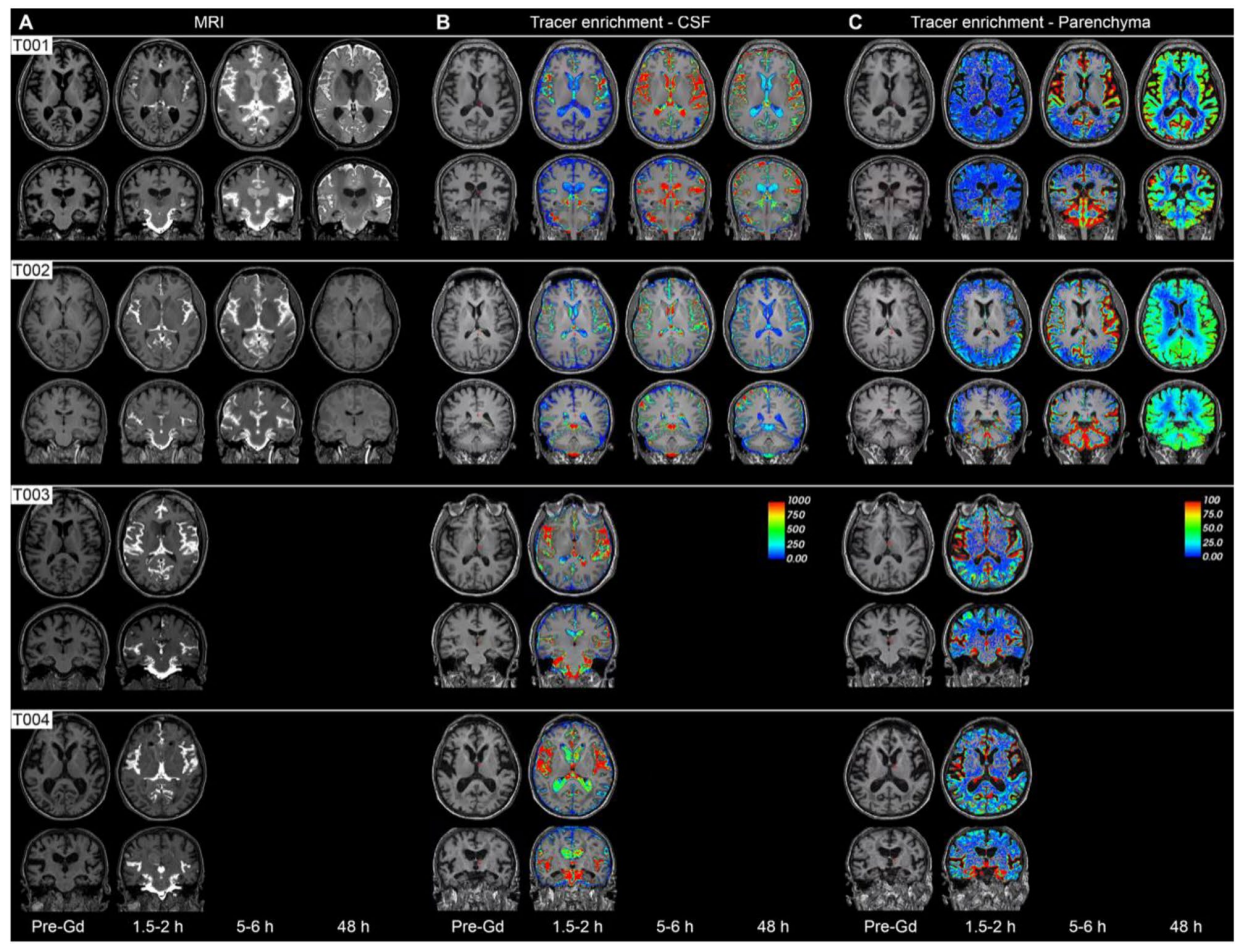
Distribution of cerebrospinal fluid (CSF) containing the tracer in the brain over time for each patient. (**A**) Panel shows T1-weighted MR images (axial and coronal planes) of the four participants before the intrathecal administration of the gadolinium-based contrast agent (Pre-Gd), and at different time points after the contrast administration (1.5-2 h, 5–6 and 48 h). (**B**) Panel shows the enrichment of the CSF measured as percentage T1 signal increase. (**C**) Panel shows the enrichment of the brain tissue measured as percentage of T1 signal increases (the CSF signal was subtracted before the calculations were performed). Scale bar for voxel intensity is shown in B and C

To homogenize the signal intensity of the different time points and compensate for eventual changes in the baseline greyscale values, the T1 signal units were divided by the T1 signal unit of a reference region of interest in the posterior part of the orbit (Additional file 1; Figure S1b), for the same time point. As the signal intensity in the CSF was 10 times higher than in the brain, we have used two different voxel intensity scales (Fig. 1B, C). For each normalized time point, the percentage of the relative increase in intensity from the previous time point was computed. Detailed information on MRI acquisition and analyses are provided in Additional file 1.

**Fig. 2:**
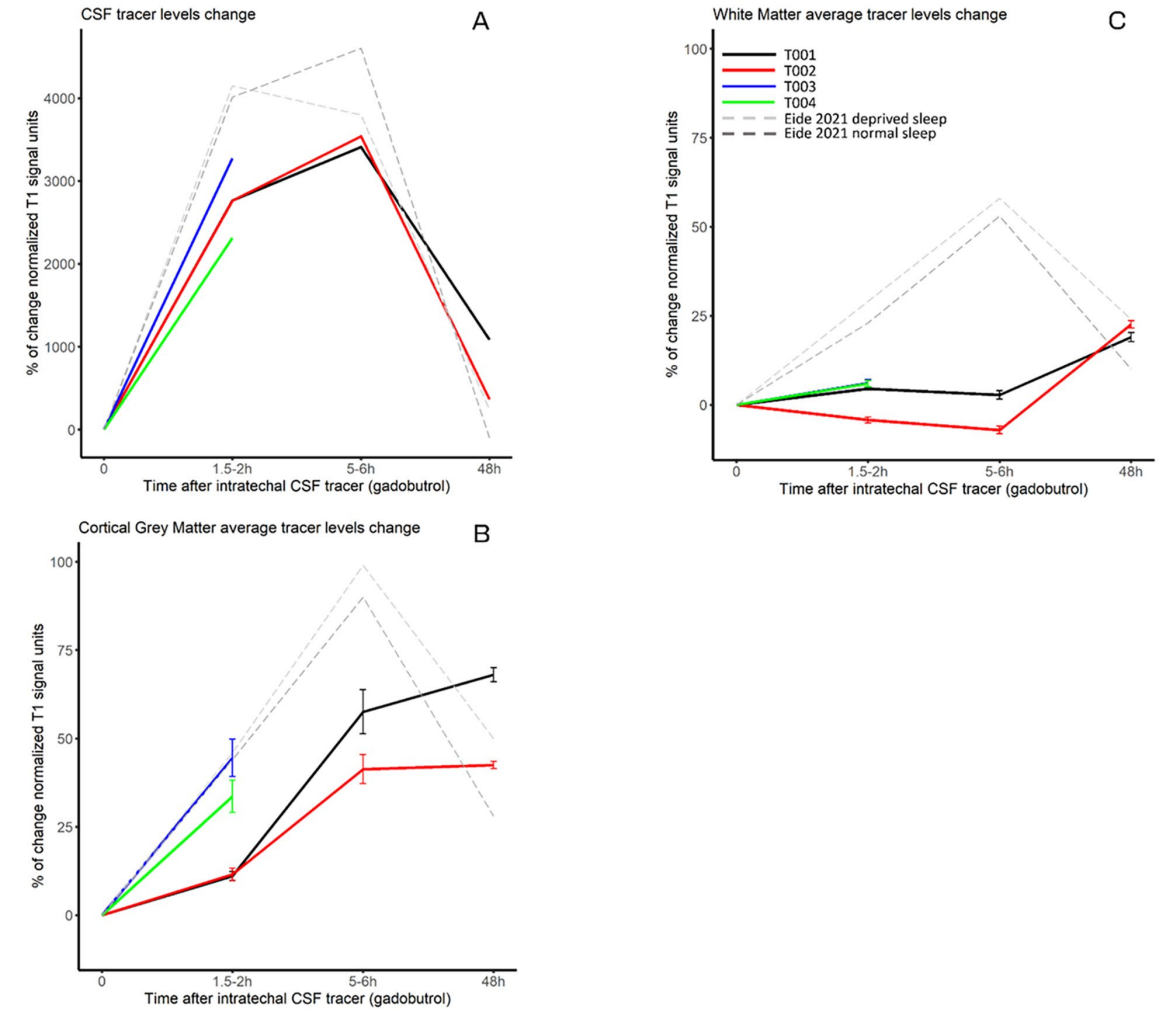
Tracer enrichment percentage of change from baseline at different time points after the contrast administration (1.5-2 h, 5–6 and 48 h) by patient. (**A**) CSF percentage of change in tracer enrichment at the region of interest in the cisterna magna. (**B**) Percentage of change of tracer enrichment in cortical grey matter, mean of the cortical regions based on FreeSurfer parcellations and standard error of the mean is shown. (**C**) Percentage of change of tracer enrichment in white matter, mean of the white matter regions based on FreeSurfer segmentations and standard error of the mean is shown. Eide et al. 2021 REF data is shown for reference

Following 1.5-2 h after intrathecal injection, the tracer was distributed within the subarachnoid cisternae as well as at spaces alongside the major cerebral arteries (anterior, middle, and posterior cerebral arteries) in all participants (Fig. 1A). Quantification of normalized MRI T1 signal intensity indicates that there was an enrichment of tracer in CSF spaces (ventricles and subarachnoid spaces) in all participants (Fig. 1B) demonstrating that the tracer has mixed well with the CSF. However, cortical tracer enrichment was higher at this time point (1.5-2 h) in participants T003 (44.5%) and T004 (33.6%) as compared to T001 (11.1%) and T002 (11.5%) (see Fig. 1 C and 2B). At 5–6 h after injection, the voxel intensity in cerebral cortex regions of participant T001 and T002 reached maximum values (57.6% and 41.4%, respectively) (see Fig. 2B). Note that these tracer enrichment values were close to those observed in participants T003 (44.5%) and T004 (33.6%) at 1.5-2 h post-tracer injection. This suggests a faster fluid flow into the brain of participants T003 and T004, with a rate of change per hour at 2 h of 22.3% (T003) and 16.8% (T004), as compared to the 5.5% and 5.7% observed in patients T001 and T002, respectively.

At 48 h after injection, there was a clear reduction in voxel intensity in the CSF of participants T001 and T002, (1086% and 364% as compared to baseline after reaching the maximum around 3500% at 5–6 h) indicating tracer clearance (Fig. 2A). In the cortex of participants T002 there was not a decrease in the signal intensity at 48 h (Fig. 2B) and patient T001 had a slight increase in tracer enrichment (from 57.6% at 5–6 h to 68.1% at 48 h, black line, Fig. 2B). In white matter there was a clear increase in the tracer enrichment only at 48 h after injection in participants T001 (19%) and T002 (22.6%) (Fig. 2C).

### Side effects after tracer injection

Following the intrathecal tracer injections, all participants experienced headaches and behavioral changes such as confusion and irritability. The side effects were most severe in participants T003 and T004, making it impossible to perform the remaining 2 subsequent MRI studies (at 5–6 h and at 48 h). Participant T001 developed nausea, vomiting, headache, and abdominal pain 3 to 4 h after contrast injection. Participant T002 developed nausea, vomiting and headache 1–2 h after contrast injection and was referred to the emergency unit with full remission of symptomatology in subsequent hours. Participant T003 showed severe side effects. Approximately 2 h after contrast injection, the participant presented temporo-spatial disorientation, hetero-aggressive outbursts, and progressive decrease in the level of consciousness followed by two tonic-clonic convulsive seizures. Head computed tomography scan ruled out intercurrent acute pathology. Twelve hours after contrast injection, the participant became asymptomatic. Participant T004 had intense vomiting 2 h after trace injection accompanied by abdominal discomfort that remitted after a few hours. Further details on side effects can be found in Additional file 1.

### Discussion and conclusions

In the present study we report the clinical side effects as well as enrichment and clearance pattern of intrathecal administrated gadobutrol in the brain of four participants diagnosed with mild to moderate cognitive impairment due to AD. All participants had unreported obstructive sleep apnea, slept poorly in the study, and presented severe side-effects related to tracer administration. In contrast to previous studies in cognitively unimpaired subjects [11, 12], we observed delayed and persistent tracer enrichment in the cortex and the white matter 48 h after injection, suggesting a poor glymphatic function in these participants.

Although preliminary, the results observed in these four cases suggest that the clearance of solutes from the brain interstitial space is decreased in participants with evidence of AD pathology, cognitive impairment, and presenting sleep alterations during a PSG. Eide and colleagues observed in non-AD participants that tracer-related signal was clearly decreasing 48 h after injection to levels lower than those observed 2 h after injection in the free sleep group (percentages of increment from baseline of 28% and 44% respectively) with a peak of signal around 5–7 h (90%) [7, 13]. Using the same procedure, we observed a delayed and prolonged tracer enrichment in the cortex and white matter. Further, there was a faster and higher initial tracer enrichment in the two participants with major side-effects that precluded them from undertaking the final two MRI scans (T003/4) as compared to the other two participants who finished the protocol (T001/2). Importantly, the participants that were discontinued were the ones that had the lowest memory performance, the lowest sleep efficiency, and the higher amyloid-beta pathology as measured by Aβ_1−42_/Aβ_1−40_.

The severe side effects observed in our study are in contrast with previous studies using the same procedure and dosing of intrathecal gadobutrol. In those studies, there was a low percentage of occurrence and mild severity of side effects in participants with CSF-related abnormalities [11, 12]. It is not clear what has caused these symptoms, however some of these side effects could be attributed to the differential amount and speed by which tracer accumulated and cleared from the brain in our participants compared to non-AD participants previously reported [7, 13]. We tentatively hypothesize that the side effects could be related to the presence of chronic sleep disorders that would impact glymphatic function and lead to the pathological accumulation of extracellular proteins characteristic of AD. In turn, this may interfere with tracer diffusion and clearance. Altered glymphatic enhancement of CSF tracer in individuals with chronic poor sleep quality has been recently reported [13], showing that tracer enrichment differed between patients with good (PSQI ≤ 5) and poor (PSQI > 5) sleep quality in a cohort of non-demented individuals (n = 44; age 42.3 ± 14.5 years), and in participants with the idiopathic normal pressure hydrocephalus dementia subtype (n = 24; age 71.0 ± 4.9 years). Interestingly, in such study no relevant age- or cognitive status-related differences in clearance patterns were observed between both groups, that had three decades of difference in age, being gadobutrol clearance mainly associated to sleep quality. Poor sleep during the study, including reduced sleep efficiency (< 70%) and occult obstructive sleep apnea, were observed in the four participants included in our study. Although we cannot draw strong conclusions on the sleep quality of the participants based only in one PSG recordings, the ubiquitous low quality of sleep in our cases during the study is higher than expected, as the prevalence of sleep disturbances in mild cognitive impairment is estimated to be 59% [14]. Focusing in OSA, a recent study using PSG in 37 MCI participants found occult OSA in 43.6% of them, being twice the prevalence of controls [15]. Although we cannot affirm that the patients of our study have a chronic sleep disorder, since a “first night effect” during the PSG could have impact their sleep quality, it is plausible that the poor sleep and OSA observed, if chronic, contributed to impaired glymphatic function as captured by the reduced contrast clearance and the observed side-effects. We performed the PSG the night before the gadobutrol injection. Therefore, no direct associations between sleep parameters and gadobutrol clearance can be drawn. A study specifically designed to explore such associations avoiding the first night effect (i.e. with two consecutive nights of PSG recordings in the 48 h after contrast injection) could have provided additional valuable information, but the side-effects observed would have surely affected the PSG recordings.

## Conclusion

This is the first study with intrathecal Gd in participants with cognitive impairment due to AD pathology. The study casts preliminary, but clinically relevant data supporting the hypothesis that sleep disturbances in participants with cognitive impairment due to AD pathology may alter protein clearance via the glymphatic system.

### Electronic supplementary material

Below is the link to the electronic supplementary material.

Supplementary Material 1

## Data Availability

Data would be available upon request to corresponding author after approval.

## Abbreviations

AD: Alzheimer’s disease
CSF: Cerebrospinal fluid
MRI: Magnetic Resonance Imaging
NREM: Non-rapid eye movement
Aβ: Amyloid beta
p-tau: Phosphorylated tau
t-tau: Total tau
Gd: Gadobutrol
DTI: Diffusion tensor imaging
CDR: Clinical Dementia Rating
MMSE: Mini-mental State Examination
RBANS: Repeatable Battery for the Assessment of Neuropsychological Status
DMI: Delayed Memory Index
PSG: Polysomnography
EEG: Electroencephalogram
EMG: Electromyogram
PLM: Periodic limb movement
REM: Rapid eye movement
OSA: Obstructive sleep apnea
AHI: Apnoea/hypopnea index
WASO: Wakefulness after sleep onset
T001: Patient 1
T002: Patient 2
T003: Patient 3
T004: Patient 4
PSQI: Pittsburgh Sleep Quality Index
