# Supplementary material for: Impairments in sleep and brain molecular clearance in people with cognitive deterioration and biological evidence of AD: a report of four cases
Source: BMC Neurol. 2023 Nov 22;23:417. doi: 10.1186/s12883-023-03460-8 (PMC10664290; doi:10.1186/s12883-023-03460-8)

**Additional file 1**

**Supplementary Methods**

*Polysomnography*

Selected participants from the cognitive tests were booked for an overnight appointment to the Sleep Clinic at the Hospital at the Hospital Universitari Mútua Terrassa for the polysomnography (PSG) study. Patients received detailed information about the recording system including positioning of electroencephalogram (EEG) and electromyogram (EMC) electrodes, nasal cannula, thermistor, and pulse-oximeter. Video-PSG studies were held in specially conditioned rooms. The electrode placement followed the 10-20 standard system, and the PSG montage included six electroencephalogram channels (EEG; F3, F4, C3, C4, O1, and O2), left and right electrooculogram (EOG); chin EMG; electrocardiogram (ECG); EMC of right and left tibialis anterior muscles; nasal pressure transducer; oronasal thermistor; snoring; thoracic and abdominal respiratory effort (inductance plethysmography); pulse oximetry-derived arterial hemoglobin oxygen saturation and pulse waveform, previously calibrated. Video-PSG recordings were performed from 22:30 PM to 6:30 AM using a PHILIPS-Respironics Alice 6 LDx polysomnographic system (Philips Respironics, PA, US) and analyzed in 30 s epochs. Sleep stages and respiratory events were scored according to the American Academy of Sleep Medicine (AASM-VIII.4B-3% desaturation <https://aasm.org/aasm-clarifies-hypopnea-scoring-criteria/>) Standard criteria. Apnea was defined as a drop in the excursion signal peak by ≥90% from the pre-event baseline measured by nasal pressure transducer measurements. Obstructive apnea was defined as the absence of airflow with continued chest wall and abdominal movement for duration of at least two breaths. Central apnea was defined as absence of inspiratory effort if: (1) the event is ≥ 10 seconds in duration, or (2) a shorter event if associated with an arousal or ≥ 3% oxygen desaturation. Hypopnea was scored if the peak signal excursion drop is ≥ 30% of pre-event baseline for at least 10 seconds. A mixed apnea was defined as an apnea that begins as central and ends in obstruction, according to changes in the chest, abdominal, and flow traces for at least 10 seconds.

*Magnetic resonance imaging (MRI) acquisition and analyses of signal intensity*

After spending the night at the Sleep clinic, patients were transferred to the Radiology Department (UDIAT-Parc Taulí Sabadell) for MRI scans and intrathecal injection of the contrast. Following a pre-contrast MRI scan, the patient was prepared for the CSF collection (5 mL) for biomarkers and proteomics analyses and the intrathecal injection of the MRI contrast (0.5 ml of 1.0 mmol/ml gadobutrol; Gadovist^R^, Bayer Pharma AG). The procedure was carried out by an expert neurologist. Correct position of the gauge needle tip in the subarachnoid space between lumbar L3/L4 or L4/L5 interspace was verified by CSF backflow from the puncture needle and CSF was collected before replacing the syringe containing the contrast diluted in 2 ml physiological serum. After the contrast injection, the patient was instructed to rotate once around the body axis on the table and kept flat until the second MRI scan was taken 1.5-2 hours after injection. The third scan was taken 5-6 hours and the last scan was taken 48 hours after injection. The participants were kept flat until the last MRI acquisition during the first day and allowed to move freely thereafter. Patients T001 and T002 were able to take the 4 MRI scans, whereas patients T003 and T004 were able to take only the first two scans due to severe side effects caused by intrathecal injection of gadobutrol.

MRI scans were acquired using a 3T MR scanner (Phillips Ingenia Elition). A standardized MR protocol was used for the acquisition, comprising of high-resolution 3D T1-weighted magnetization-prepared rapid gradient echo (MPRAGE) imaging sequence for enhanced tissue contrast, with the following settings: TR = 6.8, TE = 3.1; TI = 606.4 msec; flip angle = 8°. Voxel dimensions were 1x1x1mm and the acquisition time for each scan was 4:13 min.

To calculate tracer enrichment in the CSF, a region of interest was manually placed in the cisterna magna in T1-weighted images of MRI scans of each patient (Supplementary Fig 1A). The obtained signal intensity was then normalized (i.e., divided) to a reference value measured from the posterior part of the orbit (Supplementary Fig 1B), where the tracer cannot reach. Tracer enrichment in brain parenchyma was calculated using The FreeSurfer software version 7.3.2 (http://surfer.nmr.mgh.harvard.edu/) First, for each subject, a robust representation of the average anatomy over time was computed from all the time points available and used as unbiased reference to register all time points. T1-weighted images associated to the first time point were then processed with Freesurfer’s command “recon-all”, which segmented the brain in cortical parcels [Fischl et al. 1999a, Dale. et al. 1999] and in deep gray matter subcortical structures (including hippocampus, amygdala, caudate, putamen and ventricles) [2002, 2004b]. Thereafter all segmentations and parcellations were visually inspected for possible errors. The segmentation and parcellation obtained from FreeSurfer were used to investigate the abundance of the tracer within the brain by analyzing the increase of T1 signal intensity in all the time points. To homogenize the signal intensity of the different time points and compensate eventual changes in the baseline greyscale values, the T1 signal units were divided by the T1 signal unit of a reference region of interest, for the same time point, which was the posterior part of the orbit as detailed above (Supplementary Fig 1B). As the signal intensity in the CSF was 10 times higher than in the brain, we have used two different voxel intensity scales (Figure 1B, C). Finally, for each normalized time point, the percentage of the relative increase in intensity from the previous time point was computed.

Experimental assay to measure biomarkers in CSF

CSF samples, obtained by lumbar puncture as described above, were collected in polypropylene tubes, centrifuged at 2000 rpm for 20 min, aliquoted in propylene tubes and stored at −80 °C until biochemical analysis using standard protocols.

The CSF biomarkers analyses were performed by Catlab (<https://www.catlab.cat/en/>) and levels of Aβ1-42, Aβ1-40, total (t)-tau, and phosphorylated tau at threonine 181 (p-tau) were measured using the following Lumipulse G β-amyloid 1-40, Lumipulse G β-amyloid 1-42, Lumipulse G pTau 181 and Lumipulse G Total Tau chemiluminescent immunoassay Lumipulse® G essay kits from Fujirebio (Fujirebio Inc. Europe, Gent, Belgium). The sensitivity and detection values were used as described by the producer and local pathological cut off values were previously described in Alvarez et al. 2018.

Detailed description of side effects after tracer injection

Following the intrathecal tracer injections, all patients experienced headaches and behavioral changes such as confusion and irritability. The side effects were most severe in patients T003 and T004, preventing from completing the remaining 2 MRI studies (at 5-6h and at 48h) of the protocol. Patient T001 developed nausea, vomiting, headache, and abdominal pain 3 to 4 hours after contrast injection. Patient T002 developed nausea, vomiting and headache 1-2 hours after contrast injection and was referred to the emergency unit with full remission of symptomatology in subsequent hours. Patient T003 showed severe side effects. Approximately 2 hours after contrast injection, the patient presented temporo-spatial disorientation, hetero-aggressive outbursts, and progressive decrease in the level of consciousness. The patient was transferred to the emergency box, upon arrival had a tonic-clonic convulsive seizure and a post-critical period with alternating episodes of alertness. During his stay in the emergency box, the patient had another convulsive episode with reactive tachycardia and asynchronous eye movements. The patient received one intravenous (IV) dose of clonazepam followed by IV injection of levetiracetam. During recovery the patient was agitated with verbal aggressiveness, remained drowsy but responded to basic verbal commands. Blood analyses showed only mild leukocytosis. Head computed tomography scan ruled out intercurrent acute pathology. Twelve hours after contrast injection, the patient becomes asymptomatic. Patient T004 developed severe side effects during the second MRI scan, approx. 2 h after contrast injection. The patient was admitted to the emergency department with intense vomiting during the imaging scan (without being able to complete it). The patient also presented abdominal discomfort, without chest pain, palpitations, and other associated symptoms such as disorientation. Further analyses showed mild metabolic acidosis with no relevant alteration in chest X-ray and electrocardiogram. The patient received symptomatic treatment with serum therapy, analgesics and antiemetics with clinical improvement. Later that day the patient was discharged.

**Supplementary Fig. 1**: Manual placement of the region of interest in the cisterna magna at the cranio-cervical junction (A) and in the fat of the posterior region of the orbit (B).


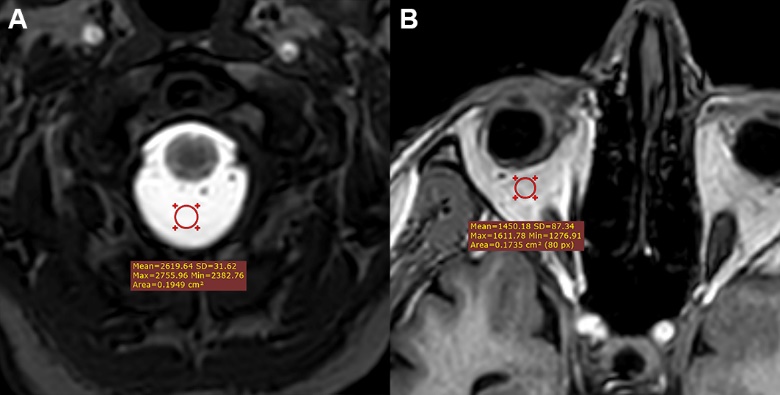

Supplement: Supplementary file 1 — Supplementary Material 1 [file 12883_2023_3460_MOESM1_ESM.docx]
